# Systematic Chromatin Accessibility Analysis Based on Different Immunological Subtypes of Clear Cell Renal Cell Carcinoma

**DOI:** 10.3389/fonc.2021.575425

**Published:** 2021-04-16

**Authors:** Shiqiang Zhang, Wenzhong Zheng, Donggen Jiang, Haiyun Xiong, Guolong Liao, Xiangwei Yang, He Ma, Jun Li, Miaojuan Qiu, Binbin Li, Chunhui Sun, Jing Zhao, Liling Wang, Jun Pang

**Affiliations:** ^1^ Department of Urology, The Seventh Affiliated Hospital, Sun Yat-sen University, Shenzhen, China; ^2^ Department of Urology, Fujian Medical University Union Hospital, Fuzhou, China; ^3^ Research Center, The Seventh Affiliated Hospital, Sun Yat-sen University, Shenzhen, China; ^4^ Maternal and Child Health Research Institute, Baoan Women’s and Children’s Hospital, Jinan University, Shenzhen, China

**Keywords:** clear cell renal cell carcinoma, the tumor microenvironment, chromatin accessibility, transcription factor, immune cell infiltration

## Abstract

**Background:**

Recent research of clear cell renal cell carcinoma (ccRCC) is focused on the tumor immune microenvironment (TIME). Chromatin accessibility is critical for regulation of gene expression. However, its role in different immunological subtypes of ccRCC based on immune cell infiltration has not been systematically studied.

**Methods:**

Five hundred thirty patient data from The Cancer Genome Atlas Kidney Renal Clear Cell Carcinoma (TCGA-KIRC) were adopted to estimate immune cell infiltration. Twenty-four types of immune cells were evaluated with single-sample Gene Set Enrichment Analysis (ssGSEA). Patients were divided into two clusters based on immune cell infiltration. Systematic chromatin accessibility analysis was conducted based on the two clusters.

**Results:**

We compared the relative expression of the immune gene signatures among 530 patients of TCGA-KIRC using ssGSEA. Overall survival (OS) analysis revealed 10 types of immune cells were significantly associated with prognosis. Patients were divided into two clusters based on 24 types of immune cell infiltration. Immune cell signals as well as PD-1/PD-L1 signal were higher in cluster 1. Among the two clusters, 2,400 differential peaks were found in TCGA-KIRC Transposase Accessible Chromatin with high-throughput sequencing (ATAC-seq) data. The distribution of differential peaks and prognosis-related immune cells in 23 chromosomes are essentially the same. There is no peak distribution downstream. The proportion of peaks upstream of the 5’ transcription start site decreases, and both sides of binding regions of the TSS 0.1-1 kb becomes smaller. Enrichment analysis of GO and KEGG of these differential peaks showed that they are remarkably related to the immune regulation in tumor microenvironment. Known motifs and *de novo* motifs were found by linking motif annotations to different peaks. Survival analysis of related motif transcription factors were prognostic. The GSEA enrichment analysis showed that high SP1 expression positively correlates with TGF-beta signaling and inflammatory response, while negatively correlates with TNF-alpha signaling *via* NFKB. High KLF12 expression negatively correlates with interferon gamma response, IL2-STAT5 signaling, TNF-alpha signaling *via* NFKB, IL6-JAK-STAT3 signaling.

**Conclusion:**

The abnormality of chromatin accessibility may play an important regulatory role in ccRCC immunity.

## Introduction

Renal cell carcinoma (RCC) is one of the most common malignant tumors in the urinary system. It is ranked top 10 most lethal tumors in both men and women in the United States (1, 2). The main pathological type is clear cell renal cell carcinoma (ccRCC), one of the most aggressive type (3), accounting for approximately 75% of all RCCs (4). With the breakthrough of immunotherapy, the tumor immune microenvironment has increasingly attracted more attention in cancer research. The tumor microenvironment heavily affects tumor progression and may affect responses to systemic therapy (3). RCC is a tumor with one of the most immune cell infiltration in pan-cancer (3), and the tumor immune microenvironment has become the focus for research in ccRCC (4, 5).

Previous studies revealed that the tumor microenvironment of ccRCC was infiltrated with high levels of different types of immune cells, which had different effects on the prognosis of ccRCC (5, 6). Single-sample GSEA (ssGSEA) was a methodology used to investigate immune cell infiltration in tumor tissues, including in ccRCC (7, 8).

Chromatin accessibility is an important epigenetic event, which is important to regulation of gene expression (9). Although encoding proteins, which only accounts 2% of the human genome, had been extensively studied, the non-coding genome and gene regulation remained to be explored and established in cancer (10). DNA regulatory elements, including enhancers, silencers, and promoters and so on, are scattered on the non-coding genome and may exert long-range influences; genes could be turned on and off by transcription factor (TF) proteins acting on these elements (10). The accessible genome accounts for ~2–3% of total DNA sequence, but captures more than 90% of regions bound by TFs (11). Chromatin state changes have been identified with tumor initiation, migration, tumor metastatic progression (12). Changes in chromatin accessibility affect the binding of TFs to their cognate genomic sequences (12). Local accessibility of chromatin is used to identify DNA regulatory elements, which tend to be relatively open due to their interactions with transcription related proteins (13).

Transposase Accessible Chromatin with high-throughput sequencing (ATAC-seq) is a method that profiles genome-wide chromatin accessibility. It uses hyperactive Tn5 transposase to assess chromatin accessibility. Sequencing reads can be used to depict accessibility and map regions of transcription factor binding and nucleosome positioning (14). Reads from a small number of cells can reflect accessible regions of chromatin through ATAC-seq (9). ATAC-seq has been used to produce comprehensive data of chromatin accessibility, and these data may serve as a fundamental resource for the cancer research community (10). Applying ATAC-seq can deepen our understanding of gene expression regulation, such as genome-wide binding sites of transcription factors and chromatin accessibility between different samples (9).

In patients with different immune infiltration status, whether they have different chromatin accessibility, and if so, whether the chromatin accessibility has a role in different immune status, have not been illustrated. In this study, we used ssGSEA based on 24 types of immune cells to compare the relative infiltration levels among TCGA-RCC patients. Using the infiltration profile of 24 types of immune cells, two immunological subtypes of patients were clustered by K-means clustering method. These two clusters of patients have differentially immune-infiltrated cells and different prognosis. To further examine changes in the chromatin accessibility between the two immunological subtypes of ccRCC, we systematically analyzed chromatin accessibility based on different immunological subtypes of ccRCC.

## Materials and Methods

### Data From TCGA Database

Molecular data of samples pathologic diagnosed with ccRCC were obtained from The Cancer Genome Atlas (TCGA) database. HTSeq transcriptome counts the data from TCGA-KIRC cohort, which contains 72 para-cancer and 539 cancer samples, and was downloaded from the Genomic Data Commons (GDC) using *TCGAbiolinks* package in R software. Corresponding clinical information of TCGA-KIRC patients was acquired from the cBioPortal (https://www.cbioportal.org/) website. After removing the para-cancer and duplicated secondary sequencing samples, 530 KIRC samples were selected and considered for further study. In the subsequent processing, gene symbol of Ensembl ID for protein-coding mRNAs was annotated by GENCODE27 and the average expression data were calculated by the *avereps* function of *Limma* package in R software when duplicated gene symbol was met. In addition, we calculated the Transcripts Per Kilobase Million (TPM) values of each gene and those genes with TPM values of <1 in over 90 percent KIRC samples served as noise signals and would be removed from further analysis. For the ATAC-Seq data, we used the raw count and normalized matrix obtained from TCGA database (10), in which the column names contain the sample IDs and each row of names corresponds to peak IDs including chromosome number, start, and end coordinates. TCGA-KIRC ATAC dataset contains 32 cancer samples and they all have matching RNA-Seq data and clinical information.

### Immune Cells Infiltration Profile Analysis

We performed ssGSEA algorithm by using the *GSVA* package in R software to calculate the signal enrichment score of 24 immune cell types in TGCA-KIRC samples (15). The ssGSEA algorithm applies background gene-set signatures by immune cell phenotype to individual samples (16, 17). Briefly, enrichment scores of the 24 immune cells are computed by 627 background gene-set signatures, and the primary enrichment score of gene signatures corresponding to target immune cell types are averaged and normalized. Then, a cell signal matrix was generated including 24 types of immune cells of each TCGA-KIRC samples. Those immune cells are involved in innate immunity [including eosinophils, neutrophils, monocytes, mast, macrophages, NK CD56 bright cells, NK CD56dim cells, dendritic cells (DCs), aDC, iDC, pDC, and natural killer (NK) cells] and adaptive immunity [such as B cells, T helper (Th), Tcm, Tem, TFH, Tgd, Th1 cells, Th17 cells, Th2 cells, Cytotoxic T cells, TReg, and CD8 T cells].

### Clustering Analysis Based on Immune Infiltrating Cells

We then performed an unsupervised clustering method (K-means) with Euclidean distance to cluster TCGA-KIRC samples based on 24 immune cell types, which could lead to distinct clinical outcomes and molecular characteristics. In this study, K-means clustering visualization was performed in R software using the *factoextra* and the *ggpubr* R packages. In addition, we calculated K-means clustering using K = 2 and K = 3 respectively, as the final result of K-means clustering is sensitive to random starting assignments, we specified nstart = 100. This means that R software will try 100 different random starting assignments and then select the best results. We further analyzed the prognosis of patients with K = 2 and K = 3, and determined the classification pattern of follow-up study. *Pheatmap* package in R software was used to observe the alterations of the global immune cell infiltrates between immune subtypes of TCGA-KIRC samples. To further analyze the molecular function of each subgroup, PD-1/PD-L1 signal reference gene-set was downloaded from MSigDB gene-set hub (http://software.broadinstitute.org/gsea/msigdb/index.jsp) and illustrated in heatmap of immune cells signals.

### Function Annotation and Gene Set Enrichment Analysis

To study the potential mechanism of immune subtypes in tumorigenesis and metastasis of KIRC patients, GSEA was performed using the *ClusterProfiler* and *org.Hs.eg.db* packages which was developed by Yu et al. (18) from Bioconductor (http://www.bioconductor.org/), and a p value less than 0.01 was considered as statistically significant. In order to analyze the biological function of TFs in TCGA-KIRC cohort, single GSEA was performed by using the *ClusterProfiler* and *org.Hs.eg.db* packages based on the median TPM value of each TF, with the 530 KIRC samples divided into high and low expression group. Reference gene-set (c2.cp.kegg.v6.2.symbols.gmt) was downloaded from MSigDB gene-set hub (http://software.broadinstitute.org/gsea/msigdb/index.jsp) and a p value less than 0.01 was considered as statistically significant.

### Difference Peaks and Annotation Analysis

Difference accessibility peaks (DAPs) between two immune subtypes were identified by *edgeR* packages in R software based on raw count ATAC-Seq data downloaded from TCGA database and a p value less than 0.05 and fold-change over 2.0 were considered as statistically significant in DAP analysis. Then, the chromosome sites of DAPs were annotated with *TxDb.Hsapiens.UCS C.hg38.knownGene, org.Hs.eg.db, ChIPseeker*, and *clusterProfiler* packages in R software. The annotation sites in chromosome of DAPs including downstream, three untranslated region (UTR), five UTR, distal intergenic, exon, intron, and promoter. Generally, the Venn diagram is a better way to present relationships of interactive sets between different data sets, but this would be hard to read when dealing with multiple data sets (over five). To address this, we used the *UpSet* package in R software in this study. The biological function of DAPs located 3,000 bp upstream and downstream of the transcription start site (TSS) were annotated with *clusterProfiler* package in R software. Finally, we ran *chromVAR*, *Biostrings*, and *BSgenome.Hsapiens.UCSC.hg38* packages in R software to obtain the sequence of DAPs and used the online tools available from Multiple EM for Motif Elicitation (MEME: http://meme-suite.org/tools/meme) to annotate and enrich DAP sequence-related motif.

### Statistical Analysis

In this study, continuous variables were described as mean and standard deviation (SD), or median and quartiles (Q), depending on the value distribution of each variable which was tested by the Shapiro-Wilk test. Categorical code variables were reported as frequencies and proportions. The statistical methodology used to compare the difference between immune subtypes of TCGA-KIRC samples included *two independent samples t-test* and *paired samples t-test* for mean values, *Mann-Whitney U-test* for median values, and *Fisher’s exact test* for frequencies and proportions variables. The correlation between two immune cell types were tested by *Spearman (Rho)* coefficients and presented as correlation matrix. In addition, we performed K-means method to cluster the 24 immune cell types and analyze the cell-cell interaction pattern between immune cells. The prognostic values of 24 immune cell types and related cluster profile were evaluated by Kaplan-Meier curve and univariate Cox regression model in R software (survival package). In this study, all statistical tests were performed in R software (Version 3.5.1; Microsoft, Redmond, WA, USA) and a p value of <0.05 (two-tailed) was considered as statistically significant.

## Results

### The Profile of Immune Cell Infiltration in ccRCC

**Figure 1:**
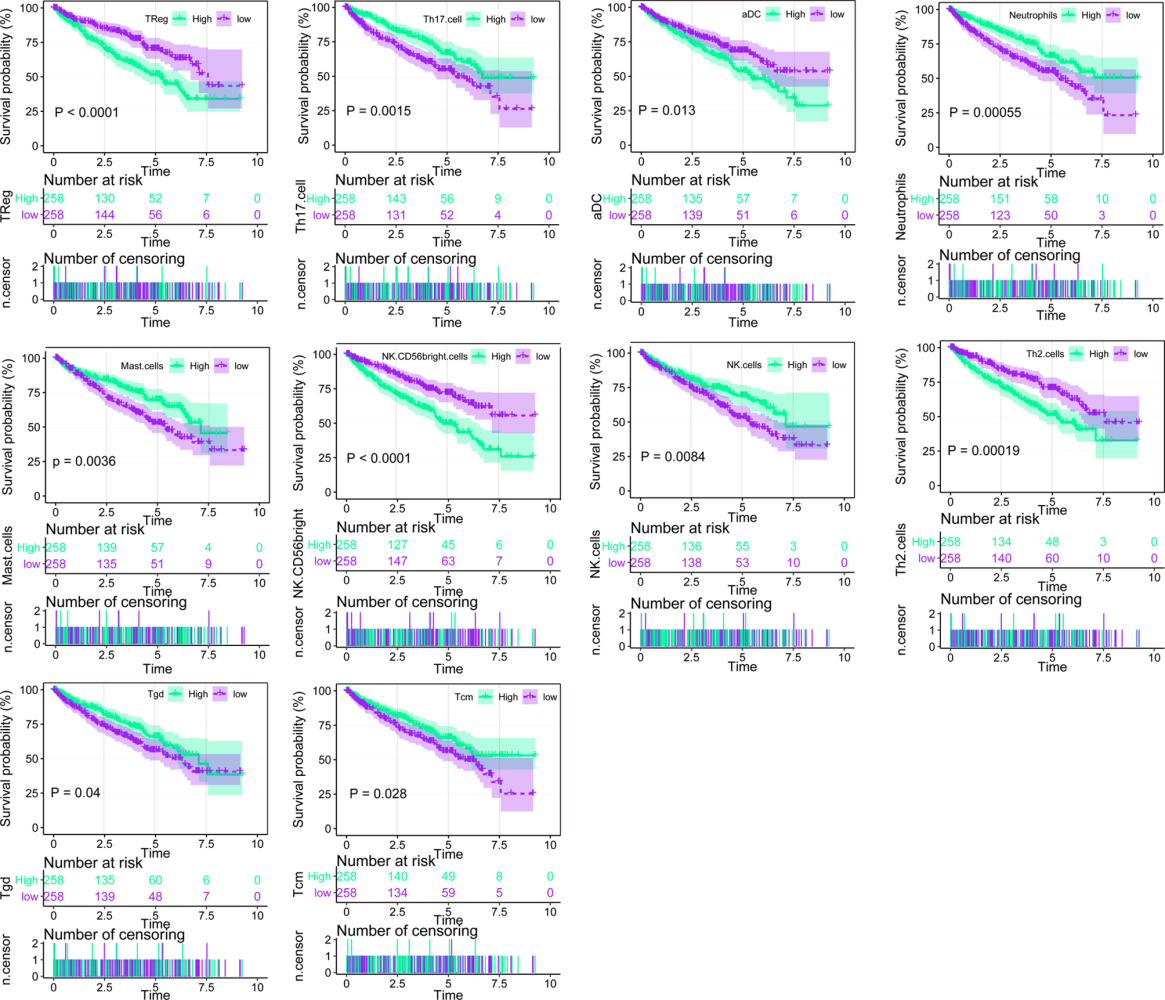
Overall survival analysis of the 24 immune cells based on median scores calculated by ssGSEA, the score higher than the median score was considered high, while lesser score was considered low.

Using ssGSEA, we identified immune infiltration of 24 types of immune cells in the 530 tumor samples of TCGA-KIRC (**Supplementary Table 1**). Overall survival analysis of every type of immune cells was performed (**Figure 1**, **Supplementary Table 2**), and we found that the presence of Treg, aDC, NK CD58 bright cells, and Th2 cells was associated with unfavorable prognosis (p < 0.001, p = 0.013, p < 0.001, and p = 0.00019), while Th 17 cells, neutrophils, mast cells, NK cells, Tgd, and Tcm were associated with favorable prognosis (p < 0.015, p = 0.00055, p < 0.0036, p = 0.0084, p = 0.04, and p = 0.0028) in ccRCC. Univariate Cox analysis of 24 types of immune cells was also conducted, showing that the presence of Th17 cells, Th2 cells, mast cells, CD56 bright NK cells, TReg, NK cells, Tgd, iDC, Th1 cells, Tcm, neutrophils, aDC, and pDC is clinically prognostic (under p < 0.05) (**Supplementary Table 3**).

**Figure 2:**
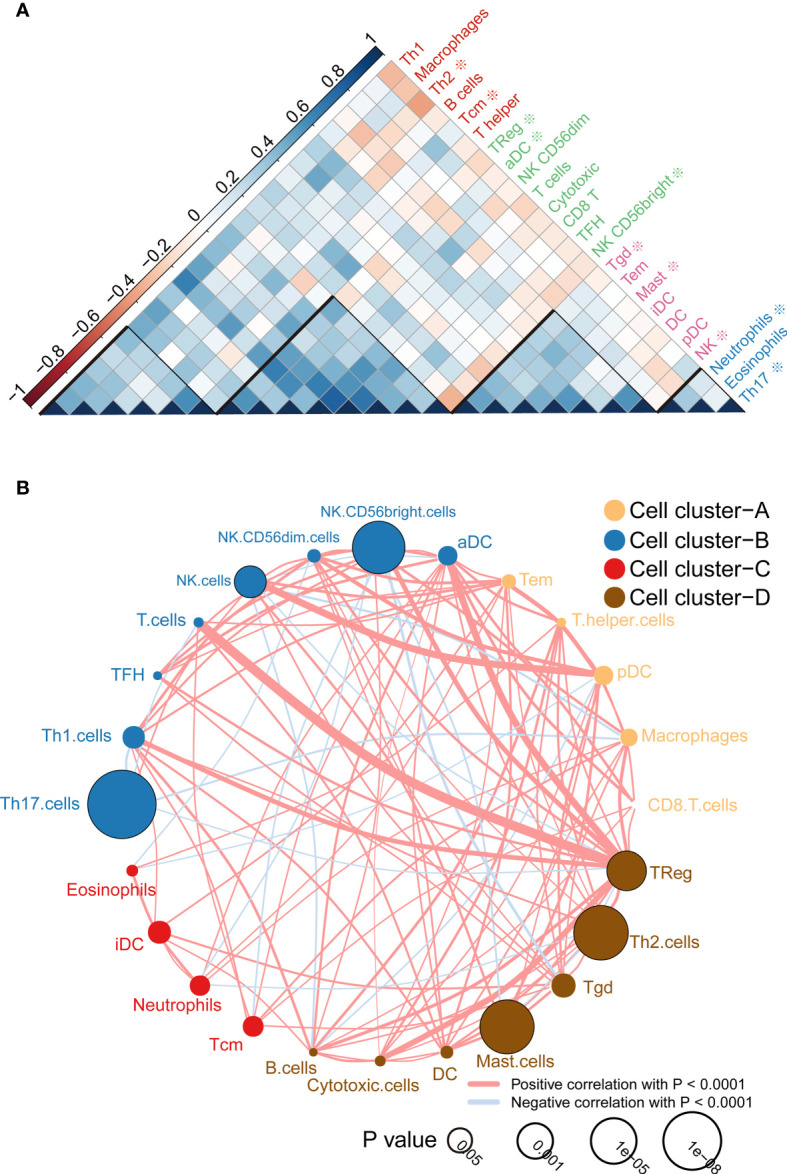
Immune cell correlation analysis and interaction network. **(A)** Correlation analysis between cells, the scale is the correlation coefficient, and it can be divided into four categories, among which those related to the prognosis of survival analysis are marked with * ; **(B)** Cell-cell interaction network, which is divided into four categories according to the K-means clustering algorithm. Line thickness represents correlation, node size represents the P value for overall survival using the Kaplan-Meier method.

According to the correlation analysis, we found that these immune cells are mainly grouped into four clusters (**Figure 2A**). Moreover, the immune cell interaction network displays a comprehensive landscape of cell cluster and their effects on the OS of patients with ccRCC, which is divided into four categories according to the K-means clustering algorithm (**Figure 2B**). From the results, it can be seen that Treg cells have the strongest connectivity with other cells and have a positive coordination effect with other cells.

### Immunological Subtypes of Patients with ccRCC According to Immune Cell Infiltration

**Figure 3:**
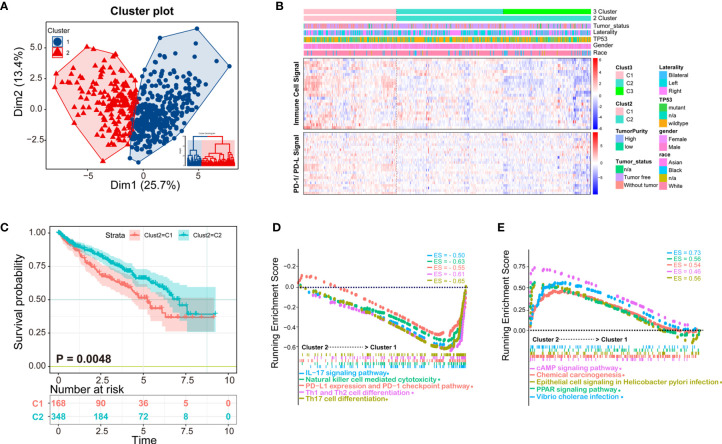
K-means clustering method identifies patients of ccRCC with distinct immune cell infiltrates. **(A)** Patients could be clustered into two groups (Cluster 1 and Cluster 2) with distinct immune cell profiles by K-means method; **(B)** The clustering heatmap of immune cell infiltrates and related clinical parameters. The heatmap of two clusters is obviously distinct, among which Cluster 1 is immune-high, and cluster2 is immune-low. The molecules related to the PD-1/PD-L1 signaling pathway are consistent with the two subtypes of patients; **(C)** The Kaplan-Meier overall survival results of two clusters, Cluster 1 has a better prognosis than Cluster 2; **(D, E)** Gene Set Enrichment Analysis (GSEA) analysis of the two clusters, in which the immune signal of Cluster 1 is higher than that of Cluster 2. The ES (enrichment score) represents the degree to which a gene set is overrepresented at the top or bottom of the ranked gene list.

Clustering of ccRCC using immune infiltration levels revealed two clusters of differentially infiltrated tumors (**Figure 3A**), namely high- and low-infiltration patients, and they were significantly distinguished among to the two clusters (**Figure 3B**). Clinical and molecular characteristics, such as race, gender, TP53 mutation, and tumor laterality had no statistical difference between the two groups (**Figure 3B**). Immune cell signal was higher in cluster 1, as well as PD-1/PD-L1 signal, which could explain why the overall survival time of cluster 1 was shorter than cluster 2 (**Figure 3C**). Patients with higher PD-1/PD-L1 signals are considered to be immunosuppressed.

According to K-means clustering analysis, there were three clusters of patients, but Cluster 2 and Cluster 3 are inseparable in prognosis thus clinically meaningless, therefore we finally selected two classification (K = 2) for further study (**Supplementary Figure 1**). Demographical and clinicopathological characteristics between Cluster 1 and Cluster 2 were shown in **Supplementary Table 4**. Under the two clusters’ classification, most immune cell scores of the 24 types of immune cells were significantly different between the two immunological subtypes of ccRCC (**Supplementary Figure 2**), except for eosinophils, iDC, neutrophils, Tcm, Tem, Tgd.

To obtain deeper insights into the function of the immune infiltration in ccRCC, we used GSEA enrichment with transcription profiling to explore altered pathways between the two clusters (**Supplementary Table 5**), and we found cluster 1 to have enriched immune-related pathways (**Figures 3D, E**). For example, Cluster 1 was enriched in IL-17 signaling pathway gene set (ES = −0.50, p = 0.003497, FDR = 0.021048), NK cell-mediated cytotoxicity gene set (ES = −0.63, p = 0.004237, FDR = 0.021048), PD-L1 expression and PD-1 checkpoint pathway (ES = −0.55, p = 0.00341297, FDR = 0.021048), Th1 and Th2 cell differentiation (ES = −0.61, p = 0.00341297, FDR = 0.021048), Th17 cell differentiation (ES = −0.65, p = 0.00349650, FDR = 0.021048) (**Figure 3D**), cAMP signaling pathway (ES = 0.46, p = 0.003571, FDR = 0.02108), chemical carcinogenesis gene set (ES = 0.54, p = 0.0042735, FDR = 0.021048), epithelial cell signaling in *Helicobacter pylori* infection (ES = 0.56, p = 0.004249, FDR = 0.021048), PPAR signaling pathway (ES = 0.56, p = 0.004267, FDR = 0.021048), and *Vibrio cholerae* infection (ES = 0.73, p = 0.001475, FDR = 0.021048) (**Figure 3E**).

### Chromatin Accessibility Analysis Based on Different Immunological Subtypes of ccRCC

**Figure 4:**
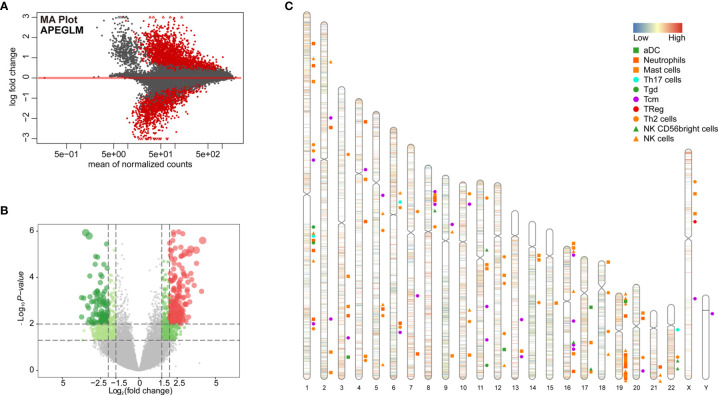
Differential peak analysis of TCGA-KIRC ATAC-seq data based on two clusters. **(A)** MA distribution map of change folds of peaks; **(B)** 2,400 differential peaks (**Supplementary Table 6**) when the corrected p-value <0.05, change folds greater than 2; **(C)** Distribution of differential peaks and prognosis-related immune cells on chromosomes.

To further examine changes in the epigenome between the two immunological subtypes of ccRCC, we analyzed chromatin accessibility with ATAC-seq data of TCGA-KIRC. There were 6 and 26 cases corresponding to cluster 1 and cluster 2 respectively. In total, 94,817 accessible locations are identified in all samples and the total peaks were shown in **Figure 4A**. In order to explore whether different chromatin accessibility leads to different immune status, we first analyzed differential peaks between two immunological subtypes, and found that there were 2,400 differential peaks under adjusted p-value <0.05, change folder <2 (**Figure 4B**). Interestingly, differential peaks and prognosis-related immune signal genes distributed across the genome in the 23 chromosomes were basically the same (**Figure 4C**), suggesting that the abnormality of chromatin accessibility plays an important regulatory role in ccRCC immunity.

**Figure 5:**
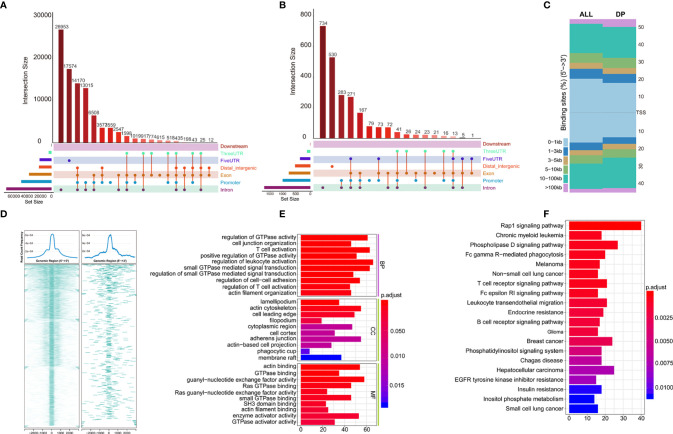
Annotations of Differential peaks. **(A)** UPSET graph shows the distribution of all peaks. No peak distribution is seen downstream, most peaks are distributed in the introns, one peak can be distributed in multiple regions at the same time (such as introns, promoter, exon, 3’-UTR), peak proportion of containing both exon and promoter is the highest. **(B)** UPSET shows the distribution of differential peaks. A higher proportion of peaks simultaneously across the 5’UTR, but the total 5’UTR area ratio decreases; **(C)** Distribution of all (ALL) and differential peaks (DP) at the transcription start site. It shows that the proportion of peaks upstream of the 5’ transcription start site decreases; **(D)** Visualization of peaks on both sides of the TSS site in the range of 0.1–1 kb, which shows differential peaks becomes smaller; **(E, F)** GO and KEGG enrichment of differential peaks (**Supplementary Table 7**).

Distribution of all peaks is shown in **Figure 5A**, and no peak distribution is seen downstream. Most of the peaks are distributed in introns, while some peaks can be distributed across multiple regions at the same time (such as introns, promoter, exon, 3’-UTR). Distribution of differential peaks (**Supplementary Table 6**) are summarized in **Figure 5B**. The distribution characteristics are similar to those of all peaks (**Figure 5A**), but some changes can be seen. For example, the ratio of peaks across the 5’UTR are simultaneously higher, but the ratio of total 5’UTR area are decreased, similar correlation can be found in the ratio of peaks simultaneously across distal intergenic regions.

Next, we analyzed total and differential peaks distribution near transcriptional start sites (TSSs). Distribution of total peaks and differential peaks near TSSs can be found in **Figure 5C**. It can be seen that the proportion of peaks upstream of TSS decreases, suggesting that the openness of these regions is reduced and binding of transcription factors are affected. Both sides of the binding regions of the TSS in the range of 0.1–1 kb are smaller (**Figure 5D**).

To further investigate the potential biological behavior of differential peaks, the *clusterProfiler* package was used to perform GO and KEGG enrichment analysis (**Figures 5E, F**, **Supplementary Table 7**). The biological processes with significant enrichment were summarized in **Supplementary Table 7**. These peaks showed enrichment of biological processes significantly related to the immune regulation in tumor microenvironment.

**Figure 6:**
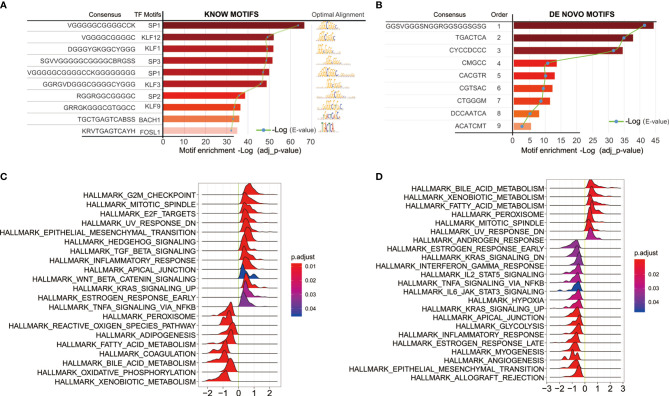
Motif annotations of different peaks **(A)** known motif, **(B)** *de novo* motif (**Supplementary Table 8**), and related motif transcription factor survival analysis results (**Supplementary Table 9**); **(C)** SP1 Single Gene GSEA Analysis of Transcription Regulators; **(D)** Single Gene GSEA Analysis of KLF12 Transcription Factors.

We next sought to link motif annotations to differential peaks for key transcriptional regulators (**Supplementary Table 8**), including known motifs (**Figure 6A**) and *de novo* motifs (**Figure 6B**). The top 10 known motifs were SP1, KLF12, KLF1, SP3, SP1, KLF3, SP2, KLF9, BACH1, FOSL1. Survival analysis of related motif transcription factor results were shown in **Supplementary Table 9** (SP1, p = 0.0144; KLF12, p = 0.0008; KLF1, p = 0.0022; SP3, p = 0.0064; KLF3, p = 0.0031; SP2, p = 0.0018; KLF9, p = 3.6899E-06; BACH1, p = 0.4767; FOSL1, p = 0.0005). The GSEA enrichment plot showed that high SP1 expression positively correlates with TGF-beta signaling and inflammatory response, while negatively correlates with TNF-alpha signaling *via* NFKB (**Figure 6C**). High KLF12 expression negatively correlates with interferon gamma response, IL2-STAT5 signaling (**Figure 6D**), and TNF-alpha signaling *via* NFKB, IL6-JAK-STAT3 signaling. These results suggest that both SP1 and KLF12 are associated with tumor immunity.

## Discussion

In the present study, we used ssGSEA method based on 24 types of immune cells to assess the infiltration rate of TCGA-KIRC, and found two different immunological subtypes (cluster 1 *vs* cluster 2), with cluster 1 having a higher immune cell signal than cluster 2, consistent with PD-1/PD-L1 signals. Overall survival analysis showed that cluster 1 had a poorer prognosis than cluster 2. We then systematically analyzed chromatin accessibility based on the two clusters. Further analysis of chromatin accessibility differences between the two clusters of patients implied that changes in chromatin accessibility may play a significant role in ccRCC immunity.

In the past decades, immunotherapy has achieved breakthroughs in various types of cancers (19). Due to dramatic heterogeneity and plasticity of the tumor immune microenvironment, and a rich existing bulk tumor sequencing data, it is still a monumental task to depict the immune cell infiltration and immune cell interactions (20–22). Many computational methods were used to infer immune cell infiltration, including CIBERSORT, xCell, MCP-counter, TIMER (20, 21, 23). In this study we used ssGSEA to quantify the immune infiltration based on 24 types of immune cells. ssGSEA is simple and can easily be adjusted, which computes an ES (enrichment score) representing the degree to which genes in a particular gene set are coordinately up- or downregulated within a single sample. Furthermore, the gene signature enrichment approach is rank-based and suitable for cross-platform evaluations (20, 21, 24).

Consistent with previous evidence, the tumor microenvironment of ccRCC was infiltrated with high levels of different immune cells, which have different effects on the prognosis of ccRCC (6–8). We found that the presence of Treg, aDC, CD58 bright NK cells, and Th2 cells were associated with unfavorable prognosis (p < 0.001, p = 0.013, p < 0.001, and p = 0.00019), while the presence of Th 17 cells, neutrophils, mast cells, NK cells, Tgd, and Tcm were associated with favorable prognosis (p < 0.015, p = 0.00055, p < 0.0036, p = 0.0084, p = 0.04, and p = 0.0028). TCGA-KIRC patients were divided into two categories by K-means cluster method based on the profiles of the 24 type immune cell infiltration. We found that patients with higher immune cell infiltration had higher PD-1/PD-L1 signal. OS benefited from low immune cell infiltration, as expected from low PD-1/PD-L1 signal. Patients with higher PD-1/PD-L1 signal represents an immune-inhibited status (19, 25)

Chromatin accessibility reflects both aggregate TF binding and the regulatory potential of a genetic locus (11). Chromatin accessibility changes are associated with tumor initiation, migration, tumor metastatic progression (12, 26, 27). ATAC-seq has emerged as one of the most widely used methods for accessing genome-wide chromatin accessibility. This method uses hyperactive Tn5 transposase, which simultaneously cuts DNA and inserts sequencing adaptors, preferentially in regions of open chromatin (9, 13). Regulation of transcription is a dynamic interaction between chromatin structure and recruitment of numerous transcription factors to DNA regulator elements, such as enhancers, upstream activator sequences, proximal promoter elements, and so on. The maintenance of accessible chromatin configurations requires binding of transcription factors to activate target genes (9). On the contrary, condensed chromatin restricts binding of transcription factors and transcriptional regulators to DNA regulator elements, which results in gene silencing (9). ATAC-seq could provide meaningful insight into the profile of chromosome accessibility.

Although there was significant difference in prognosis of the two clusters of TCGA-KIRC, the role of chromatin accessibility in ccRCC immunity has not been fully explored. In our study, we attempted to explore if chromatin accessibility changes are associated with tumor immunity using ATAC-seq data of TCGA-KIRC. We analyzed the distribution of peaks in the chromosomes, and found that there were 2,400 differential peaks under the adjusted p-value <0.05, change folder <2 (**Figure 4B**). Differential peaks and prognosis-related immune signal cells were distributed across the genome in a similar manner, suggesting that the abnormality of chromatin accessibility plays an important regulatory role in ccRCC immunity. The different peaks are distributed across the genome in a similar manner among all peaks, for example, there is no peak distribution downstream, indicating that the open chromatin area which plays a role in transcription regulation is mainly located upstream of the genes. But some changes can be seen, for example, the proportion of peaks upstream of the 5 ‘transcription start site decreases, and both sides binding regions of the TSS 0.1–1 kb becomes smaller, suggesting that the accessibility of these regions is reduced and transcription factor binding are affected. As expected, GO and KEGG enrichment analysis of these different peaks showed remarkably related to the immune regulation in tumor microenvironment, for example, T cell activation and regulation of T cell activation in GO terms, Fc gamma R-mediated phagocytosis, T cell receptor signaling pathway, Fc epsilon RI signaling pathway, and B cell receptor signaling pathway in KEGG pathways.

Previous evidence suggests that changes in chromatin accessibility affect the binding of transcription factors (TFs) to their cognate genomic sequences (12). The maintenance of accessible chromatin configurations requires binding of transcription factors to activate target genes (9). Considering the role of transcription factors in open chromatin, key transcription factors were found by linking motif annotations to different peaks. The top 10 known motifs were SP1, KLF12, KLF1, SP3, SP1, KLF3, SP2, KLF9, BACH1, and FOSL1. Overall survival analysis of related motif transcription factor showed that these factors had impacts on the patient’s prognosis (**Supplementary Table 8**; SP1, p = 0.0144; KLF12, p = 0.0008; KLF1, p = 0.0022; SP3, p = 0.0064; KLF3, p = 0.0031; SP2, p = 0.0018; KLF9, p = 3.6899E-06; BACH1, p = 0.4767; FOSL1, p = 0.0005). The enrichment plot of GSEA analysis of high SP1 expression shows positive correlation with TGF-beta signaling and inflammatory response, and negative correlation with TNF-alpha signaling *via* NFKB. High KLF12 expression negatively correlates with interferon gamma response, IL2-STAT5 signaling, and TNF-alpha signaling *via* NFKB, IL6-JAK-STAT3 signaling. These results suggest that both SP1 and KLF12 are associated with tumor immunity.

SP1 is one ubiquitous TF from the Sp/Kruppel-like family (KLF) TFs. It is involved in numerous cellular processes, including cell differentiation, cell growth, apoptosis, immune responses, response to DNA damage, and chromatin remodeling (28–32). The role of SP1 in RCC had also been investigated before. SP1 could bind to the promoter region of SNHG14 to upregulate its expression to promote migration and invasion of ccRCC (33, 34). Previous study also demonstrated that dephosphorylated Sp1 was more tightly associated with chromatin than its phosphorylated counterparts from either resting or mitotic cells (30), which suggested that the status of phosphorylation would affect the chromatin accessibility. Studies have shown that KLF12 regulates proliferation of cancer cell lines. Overexpression of KLF12 in endometrial and lung cancer cell lines correlated with increased cellular proliferation, decreased apoptosis, and increased *in vivo* tumor growth (35). Interestingly, one study indicated that KLF12 could regulate NK cell proliferation in mouse, indicating that KLF12 plays a major role in immunity (35). However, this study is not devoid of limitations. First, we have not found other datasets such as GEO datasets that includes ATAC-seq data, so there was lack of validation cohort to prove our finding. Second, all of these findings was not supported with at any explanation of possible mechanisms.

## Conclusion

In summary, this study revealed that there were two different immunological subtypes (cluster 1 *vs* cluster 2) based on the 24 types of immune cells used to assess the immune infiltration of TCGA-KIRC, and that cluster 1 had higher immune cell signal than cluster 2, consistent with the PD-1/PD-L1 signal. Survival analysis found cluster 1 had a poorer prognosis than cluster 2. Systematically analyzed chromatin accessibility based on two clusters found that the differential peaks and prognosis-related immune signal cells are similarly distributed in the chromosomes. Further analysis of key transcription factors between the two clusters revealed that SP1, KLF12, KLF1, SP3, SP1, KLF3, SP2, KLF9, BACH1, FOSL1, and more may play an important role in these two different immunological subtypes. Further molecular biology experiments *in vivo* and *in vitro* are needed to investigate the mechanism of different immune status and the exact role of transcription factors in chromatin accessibility in ccRCC.

## Data Availability Statement

Publicly available datasets were analyzed in this study. This data can be found here: The Cancer Genome Atlas (https://portal.gdc.cancer.gov/) - TCGA-KIRC.

## Author Contributions

SZ and WZ designed the study and designed the research. DJ, HX, GL, and XY collected the data. HM, JL, MQ, BL, CS, and JZ completed the data analysis and interpretation. LW, JP, SZ, and WZ drafted and revised the article for important intellectual content. All authors contributed to the article and approved the submitted version.

## Funding

This study was supported by Research Start-up Fund of the Seventh Affiliated Hospital, Sun Yat-sen University, the National Natural Science Foundation of China (81772754), Research Project of Shenzhen Health Family Planning System (SZBC2018001), Shenzhen Science and Technology Program (JCYJ20190809164617205), and the Key Research Project of Natural Science Foundation of Guangdong Province, China (2017A03038009).

## Conflict of Interest

The authors declare that the research was conducted in the absence of any commercial or financial relationships that could be construed as a potential conflict of interest.
