# Supplementary figures and images for: Systematic Chromatin Accessibility Analysis Based on Different Immunological Subtypes of Clear Cell Renal Cell Carcinoma
Source: Front Oncol. 2021 Apr 16;11:575425. doi: 10.3389/fonc.2021.575425 (PMC8085385; doi:10.3389/fonc.2021.575425)

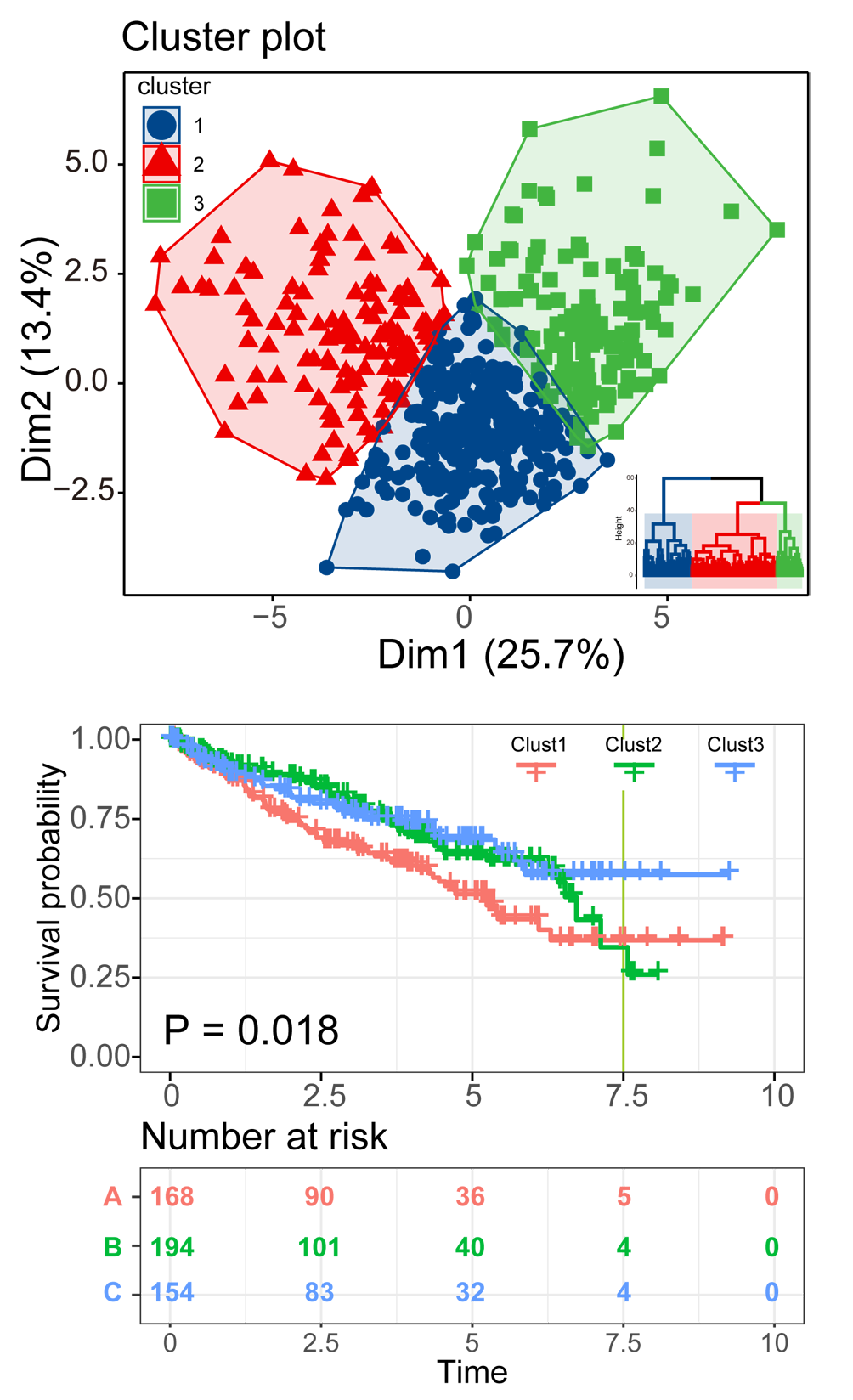

Supplement: Supplementary file 10 [file Image_1.tif]

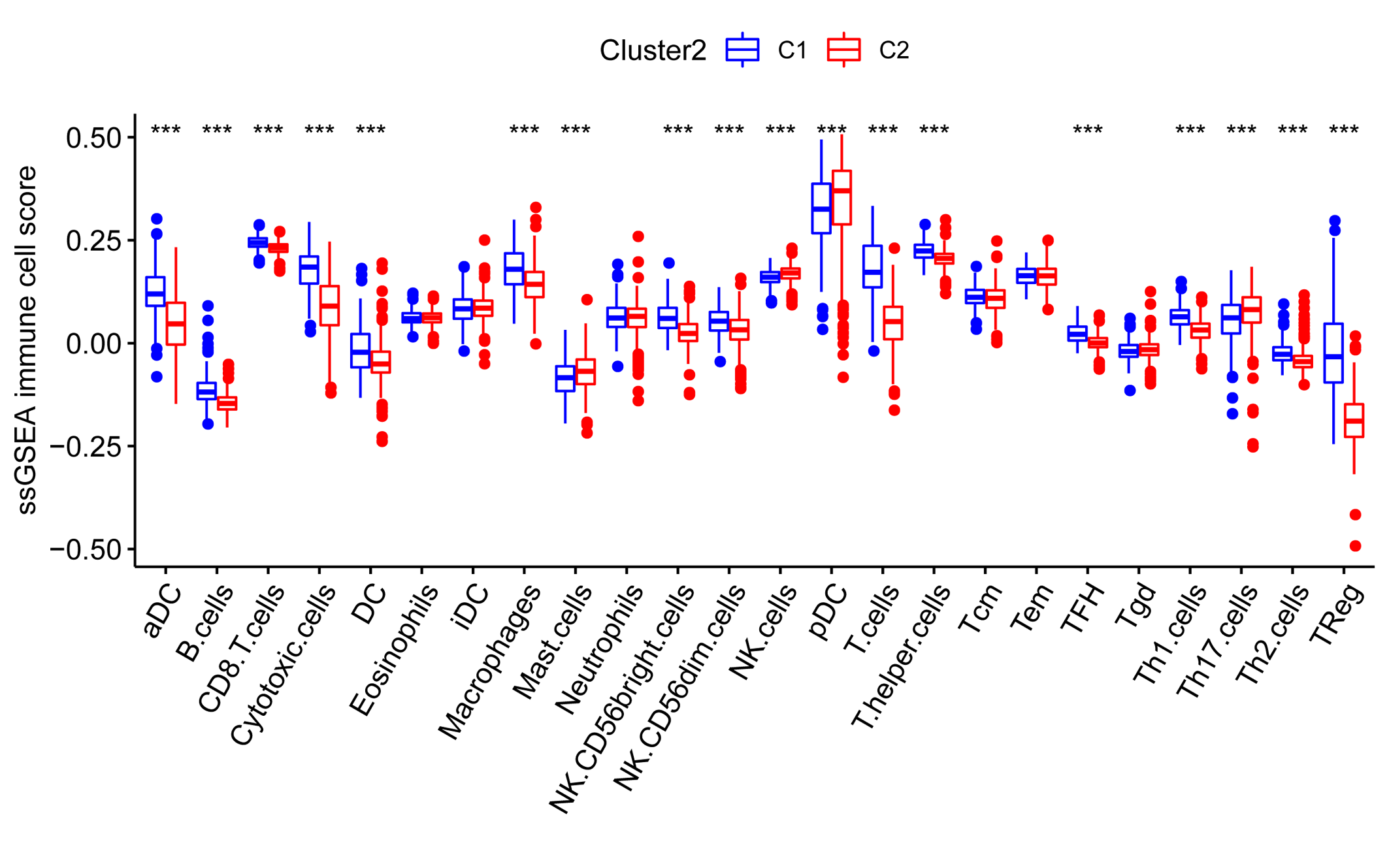

Supplement: Supplementary file 11 [file Image_2.tif]
